# Optimizing Antioxidant Activity and Phytochemical Properties of Peppermint (*Mentha piperita* L.) by Integrative Application of Biofertilizer and Stress-Modulating Nanoparticles under Drought Stress Conditions

**DOI:** 10.3390/plants12010151

**Published:** 2022-12-28

**Authors:** Ali Ostadi, Abdollah Javanmard, Mostafa Amani Machiani, Karim Kakaei

**Affiliations:** 1 Department of Plant Production and Genetics, Faculty of Agriculture, University of Maragheh, Maragheh 55181-83111, Iran; 2 Department of Chemistry, Faculty of Science, University of Maragheh, Maragheh 55181-83111, Iran

**Keywords:** drought stress, essential oil quality, medicinal and aromatic plants, menthol, peppermint, secondary metabolites

## Abstract

Drought stress (DS) negatively affects plant growth, productivity, and quality in semi-arid and arid regions. Nowadays, application of biofertilizers and stress-modulating nanoparticles (NPs) improves plant performance under stressful conditions. The study evaluated the impacts of arbuscular mycorrhizal fungi (Myco-Root) and TiO_2_ NPs on the nutrient uptake, dry yield, essential oil (EO) productivity, and EO quality of peppermint (*Mentha piperita* L.) under different irrigation regimes. The treatments included three irrigation regimes containing irrigation after 20% (I_20_, well-watered), 40% (I_40_, mild DS), and 60% (I_60_, severe DS) maximum allowable depletion (MAD) percentage of the soil’s available water as well as four fertilizer sources contain no fertilization (control), Myco-Root biofertilizer, TiO_2_ NPs, and an integrative application of Myco-Root + TiO_2_ NPs. The results demonstrated that the highest (195.72 g m^−2^) and the lowest dry yield (78.76 g m^−2^) of peppermint was obtained in well-watered conditions with integrative application of Myco-Root + TiO_2_ NPs and severe drought stress (I_60_) without fertilization, respectively. The dry yield of peppermint was reduced by 27.7 and 53.4% in mild (I_40_) and severe drought stress (I_60_), respectively. The maximum EO content (1.49%) and EO yield (2.30 g m^−2^) was recorded in mild drought stress (I_40_) treated with Myco-Root + TiO_2_ NPs. Based on the GC-MS and GC-FID analysis, 29 constituents were identified in peppermint EO, with the major constituents being menthol (38.99–52%), menthone (12.72–20.13%), 1,8-cineole (6.55–7.84%), and *neo*-menthol (3.14–4.52%), respectively. The maximum content of menthol, 1,8-cineole, and *neo*-menthol was obtained under mild drought stress (I_40_) fertilized with Myco-Root + TiO_2_ NPs. The results indicate that the integrative application of Myco-Root + TiO_2_ NPs could be used as an alternative method of using chemical fertilizers in sustainable agricultural systems for improving the EO quantity and quality of peppermint grown under drought stress conditions.

## 1. Introduction

Worldwide demand for the cultivation and usage of medicinal and aromatic plants (MAPs) has increased due to their pharmaceutical and therapeutic potentials [1]. Peppermint (*Mentha piperita* L.), as one of the MAPs belonging to the Lamiaceae family, is a natural hybrid derived from *Mentha aquatica* L. and *Mentha spicata* L. [2]. The global demand for peppermint increases day by day due to medicinal and flavoring properties and the use of this plant in cosmetic, pharmaceutical, and perfumery industries. Peppermint, as a traditional and folkloric medicine, is used for the treatment of colic, common cold, headache, sinusitis, sore throats, muscle aches, bronchitis, joint pain, gallstones, tuberculosis, and skin problems [3,4]. The volatile essential oil (EO) distilled from aerial parts of peppermint has antioxidant, antibacterial, antiviral, and antifungal activities [3]. The medicinal properties of peppermint are attributed to menthol and menthone compounds. It has been reported that the main EO constituents of peppermint include menthol, 1,8-cineoile, menthone, *neo*-menthol, *δ*-terpineol, and menthofuran [5].

The average forecasted temperature increasing by 0.75–4 °C by 2100 [6]. In Iran, the mean annual rainfall is 250 mm (70% less than the world average). The accelerating global warming decreases soil water resources and increases drought stress (DS), which negatively affect crop productivity and quality [6,7]. The plant’s productivity declined by 13–94% in DS conditions based on the plant’s type and also the severity and duration of DS [8]. DS is one of the main environmental stresses [9] caused by a reduced photosynthetic rate, production of reactive oxygen species (ROS), imbalance in absorption and loss of water, stomatal closure and oxidative damage into the chloroplast, increased photorespiration, ATP synthesis obstructed, and loss water use efficiency, which lead to decreasing plant productivity [10]. In MAPs, secondary metabolites play a main role in alleviating, adaptation, and increasing the resistance of plants against adverse environmental conditions [11]. Increasing the synthesis and production of secondary metabolites such as EOs, alkaloids, phenolic, etc. in medicinal plants reduced the damage of ROS compounds under stressful conditions [12,13]. The results of previous studies on different MAPs, including thyme (*Thymus vulgaris* L.) [11], peppermint [14], sage (*Salvia officinalis* L.) [15], and oregano (*Origanum vulgare* L.) [16], showed that the concentration of secondary metabolites increased under DS conditions. In addition, the nutrient availability decreased under DS conditions due to less soil moisture and reduction of diffusion and mass flow [17]. To achieve higher crop productivity in these conditions, farmers use more chemical inputs, which increase production cost and have negative impacts on the soil’s beneficial microorganisms [18]. It is worth noting that the excessive application of chemical fertilizers decreased the quality of MAPs through reducing the bioactive compounds of these plants [19]. Therefore, replacing the eco-friendly strategies for increasing plant performance and decreasing the negative effects of chemical fertilizers under DS conditions seems necessary. The application of biological fertilizers (bio-fertilizers) and stress-modulating nanoparticles (NPs) compounds has a high potential to improve the quantity and quality of plant performance under DS conditions.

The application of arbuscular mycorrhizal fungi (AMF) as a bio-fertilizer affects plants’ performance under unstressed and stressful conditions by increasing absorption of inorganic nutrients and water uptake, relative water content of plant cells, and enhancing photosynthesis rate [20,21,22,23]. One of the other benefits of AMF to plants is changing the concentration of primary and secondary metabolites, which play a main role in increasing the tolerance of plants in face with stressful conditions. The results of previous studies on different plants such as thyme (*Thymus daenensis* Celak, *Thymus vulgaris* L.) [24], dragonhead (*Dracocephalum moldavica* L.) [25], and basil (*Ocimum tenuiflorum* L.) [26], showing that the symbiotic association of AMF with host plants improved the EO productivity and quality of these plants.

Nanotechnology is one of the novel and creative approaches in the agricultural sector for modulating the negative impacts of stressful conditions and decreasing the hazardous effects of chemical fertilizers [27]. Nanoparticles (NPs) are described as materials with dimensions of 1 to 100 nm that easily penetrate to plants. Nanomaterials have unique electrical, thermal, and physicochemical properties such as high solubility, high specific surface area, extreme pore size, particle morphology, controlled release, smart delivery, and being catalytic [28]. Application of stress alleviated nanoparticles to reduce detrimental effects of abiotic stresses can be a cost-effective strategy to improve plant growth and productivity. Titanium dioxide (TiO_2_) NPs, as a stress-modulating NP, can promote plant growth, especially under stressful conditions, through improving root growth and nutrient absorption, regulating antioxidant enzyme activities, transforming inorganic nitrogen to organic nitrogen in the form of protein, amplifying chlorophylls (a and b) biosynthesis, increasing the stability of the cell membrane, and enhancing photosynthetic carbon assimilation by more activation of Rubisco activase [27,28,29,30]. Earlier studies reported the positive impact of TiO_2_ NPs on the growth, yield performance, and especially, productivity of biochemical components of MAPs. For example, Ahmad et al. [31] noted that the foliar spraying of TiO_2_ NPs on peppermint increased the dry weight, EO content, EO yield, and main constituents such as menthol, menthone, and menthyl-acetate. Golami et al. [32] noted that foliar spraying of TiO_2_ NPs affected the EO productivity and quality of rosemary (*Rosmarinus officinalis* L.) through increasing the major EO constituents such as *α*-pinene, camphene, limonene, 2-bornanone, and bornyl acetate.

Due to decreasing the efficiency of chemical fertilizers in drought stress conditions as well as the negative impacts of the synthetic fertilizers on the bioactive compounds of MAPs, the application of sustainable and eco-friendly fertilizers and new anti-stress nanoparticles is necessary. Among different methods of TiO_2_ NPs application (seed coating, root application, and foliar spraying) as stress-modulating nanoparticles in plants, the foliar spraying of these nanoparticles is the most effective method for improving plant performance under stressful conditions [27]. It is worth noting that in the foliar spraying, a lower concentration of these nanoparticles is needed. In contrast, in the root application of TiO_2_ NPs, higher concentrations are needed to improve plant growth, and the risk of its toxicity for plants in this condition is greater than foliar spraying [27]. The literature is scant about the integrative application of bio-fertilizers along with stress-modulating NPs. We hypothesis that i) the integrative application of Myco-Root + TiO_2_ NPs enhanced the nutrient concentration, ii) integrative application of Myco-Root + TiO_2_ NPs promotes photosynthesis pigments and productivity, and iii) integrative application of Myco-Root + TiO_2_ NPs improves essential oil quantity and quality of peppermint. Therefore, the study evaluated the separate and integrative application effects of Myco-Root (AMF) as a bio-fertilizer and TiO_2_ as stress-modulating NPs on the morphological, physiological, and phytochemical characteristics of peppermint under drought stress conditions.

## 2. Results

The analysis of variance (ANOVA) results showed that the AMF colonization, nutrient content, dry yield, essential oil (EO) content, EO yield, chlorophylls (a, b, and total), relative water content (RWC), proline, antioxidant enzymes (SOD, APX, and GPX) activity, total phenolic and flavonoid content, and net income of peppermint was impacted significantly by interaction of irrigation regimes × fertilizers application. In addition, the plant height, canopy diameter, and carotenoid content were significantly impacted by main factor effects, and the interaction of irrigation regimes × fertilizers application did not significantly impact the three mentioned traits. Additionally, the malondialdehyde (MDA) content was significantly affected by irrigation regimes (Table 1 and Table 2).

### 2.1. AMF Colonization

**Figure 1:**
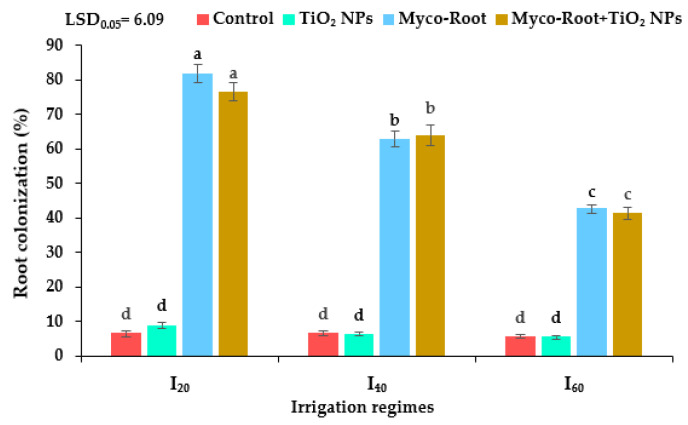
The effects of different irrigation regimes and fertilizer sources on the root colonization of peppermint. I_20_, I_40_, and I_60_ indicating well-watered, mild, and severe drought stress, respectively. Different letters indicate significant differences at the 5% level according to LSD’s test.

The highest root colonization (81.83%) was achieved in well-watered conditions (I_20_) treated with Myco-Root, followed by integrative application of Myco-Root + TiO_2_ NPs. The AMF inoculation rate decreased by 19.7 and 45.4% in mild (I_40_) and severe drought stress (I_60_), respectively (Figure 1).

### 2.2. Nutrient Content

The highest content of N, P, and K in peppermint was obtained in I_20_ (well-watered conditions) fertilized with Myco-Root + TiO_2_ NPs. Furthermore, the lowest content of the three mentioned elements was observed in I_60_ (severe DS) without fertilization. On average, the content of N, P, and K decreased by 12.2, 13.9, and 12% in I_40_ and decreased by 25.6, 22.3, and 24.2% in I_60_, respectively, when compared with well-watered conditions. Additionally, the content of the three mentioned nutrients was enhanced by 16, 19.1, and 15.81% after application of Myco-Root + TiO_2_ NPs (Table 3).

### 2.3. Plant Height

**Figure 2:**
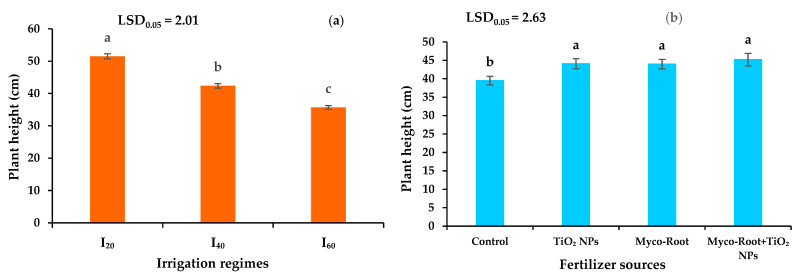
The effects of different irrigation regimes (**a**) and fertilizer sources (**b**) on the plant height of peppermint. I_20_, I_40_, and I_60_ indicating well-watered, mild, and severe drought stress, respectively. Different letters indicate significant differences at the 5% level according to LSD’s test.

Among different irrigation levels, the highest (51.49 cm) and the lowest (35.70 cm) plant height of peppermint was measured in I_20_ and I_60_, respectively. The height of peppermint was reduced by 17.7 and 30.7% in I_40_ and I_60_ (Figure 2a). In addition, the application of Myco-Root, TiO_2_ NPs, and integrative application of Myco-Root + TiO_2_ NPs enhanced the height of peppermint by 11.4, 11.6, and 14.4%, respectively, when compared with control (Figure 2b).

### 2.4. Canopy Diameter

**Figure 3:**
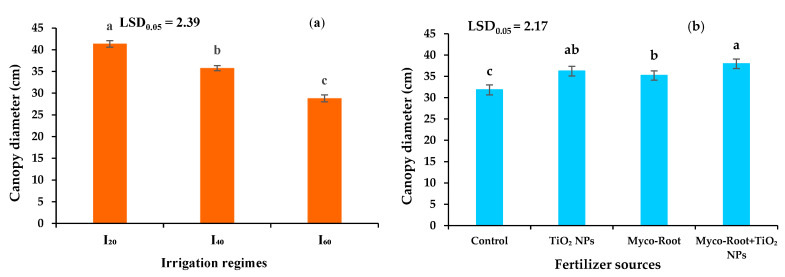
The effects of different irrigation regimes (**a**) and fertilizer sources (**b**) on the canopy diameter of peppermint. I_20_, I_40_, and I_60_ indicating well-watered, mild, and severe drought stress, respectively. Different letters indicate significant differences at the 5% level according to LSD’s test.

Application of Myco-Root, TiO_2_ NPs, and integrative application of Myco-Root + TiO_2_ NPs enhanced the canopy diameter of peppermint by 10.6, 13.8, and 19.2%, respectively, when compared with control (Figure 3b). Among different irrigation levels, the highest (41.36 cm) and the lowest (28.81 cm) canopy diameter of peppermint was measured in I_20_ and I_60_, respectively. The canopy diameter of peppermint was reduced by 13.5 and 30.3% in I_40_ and I_60_ (Figure 3a).

### 2.5. Dry Yield

Integrative application of Myco-Root + TiO_2_ NPs under well-watered conditions produced the highest dry yield of peppermint (195.72 g m^−2^), while the lowest dry yield (78.76 g m^−2^) was obtained in severe drought stress without fertilization. The peppermint dry yield was reduced by 27.7 and 53.4% in mild (I_40_) and severe DS (I_60_). Application of Myco-Root, TiO_2_ NPs, and integrative application of Myco-Root + TiO_2_ NPs increased the dry yield of peppermint by 8.7, 12.1, and 20.1%, respectively, when compared with control (Table 4).

### 2.6. Essential Oil Content and Yield

The maximum EO content (1.49%) and yield (2.30 g m^−2^) of peppermint was recorded in mild DS (I_40_) treated by Myco-Root + TiO_2_ NPs. The EO productivity under mild DS (I_40_) was 22.3% higher than non-stress conditions. Also, integrative application of Myco-Root + TiO_2_ NPs increased the EO content and EO yield of peppermint by 11.7 and 35.1%, respectively (Table 4).

### 2.7. Essential Oil Constituents

Based on the GC-MS and GC-FID analysis, 29 constituents were identified in peppermint EO, with the major constituents being menthol (38.99–52%), menthone (12.72–20.13%), 1,8-cineole (6.55–7.84%), and *neo*-menthol (3.14–4.52%), respectively. The maximum content of menthol, 1,8-cineole, and *neo*-menthol was obtained in mild DS (I_40_) fertilized with Myco-Root + TiO_2_ NPs. Additionally, the maximum content of menthone was observed in I_20_ conditions treated with TiO_2_ NPs. On average, integrative application of Myco-Root + TiO_2_ NPs enhanced the content of menthol, 1,8-cineole, and *neo*-menthol by 7.4, 8.7, and 26.7% in comparison with control (no fertilization) (Table 5).

### 2.8. Water Use Efficiency (WUE)

The highest WUE (177.77 g m^−3^) in peppermint was recorded in mild drought stress (I_40_) fertilized with Myco-Root + TiO_2_ NPs. Under mild and severe DS, the WUE of peppermint decreased by 3.6 and 6.8% in comparison with well-watered conditions. Additionally, application of Myco-Root, TiO_2_ NPs, and integrative application of Myco-Root + TiO_2_ NPs was enhanced WUE by 9.4, 11.6, and 18.5%, respectively (Table 4).

### 2.9. Chlorophylls Content

Integrative application of Myco-Root + TiO_2_ NPs under I_20_ irrigation regimes produced the maximum content of Ch_a_, Ch_b_, and Ch_total_. In contrast, the lowest content of the three mentioned traits was obtained in severe DS (I_60_) without fertilization. The content of Ch_a_, Ch_b_, and Ch_total_ decreased by 39, 22.2, and 35.2% in mild DS (I_40_) and decreased by 77.6, 68.3, and 75.4% in severe DS (I_60_), respectively. Moreover, integrative application of Myco-Root + TiO_2_ NPs enhanced three photosynthesis pigments by 39.2, 55.9, and 43.3%, respectively (Table 6).

### 2.10. Carotenoid Content

**Figure 4:**
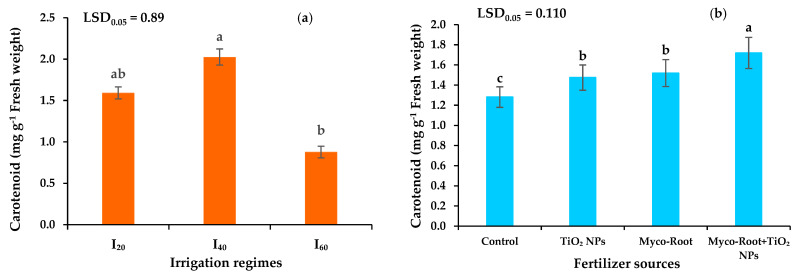
The effects of different irrigation regimes (**a**) and fertilizer sources (**b**) on the carotenoid concentration of peppermint. I_20_, I_40_, and I_60_ indicating well-watered, mild, and severe drought stress, respectively. Different letters indicate significant differences at the 5% level according to LSD’s test.

Among different irrigation levels, the maximum and minimum carotenoid concentration of peppermint was measured in I_40_ and I_60_, respectively. The carotenoid concentration was enhanced by 27% in I_40_ and reduced by 44.6% in I_60_ (Figure 4a). Application of Myco-Root, TiO_2_ NPs, and integrative application of Myco-Root + TiO_2_ NPs enhanced the carotenoid concentration of peppermint by 18.8, 14.8, and 34.4%, respectively (Figure 4b).

### 2.11. Relative Water Content (RWC)

The RWC of peppermint was the highest (90.12%) in well-watered conditions (I_20_) fertilized with Myco-Root + TiO_2_ NPs. Furthermore, the lowest RWC content (61.13%) was obtained in severe DS (I_60_) without fertilization. Under mild and severe DS conditions, the RWC content decreased by 10.6 and 21.1% in comparison with well-watered conditions. Additionally, application of Myco-Root, TiO_2_ NPs, and integrative application of Myco-Root + TiO_2_ NPs enhanced RWC content by 12.3, 8.5, and 13.7%, respectively (Table 6).

### 2.12. Proline

The maximum proline concentration (2.07 µmol g^−1^ fresh weight) was obtained under severe drought stress conditions following application of Myco-Root + TiO_2_ NPs. The proline concentration was enhanced by 124.6 and 188.5% in mild and severe drought stress conditions, respectively. Moreover, application of Myco-Root, TiO_2_ NPs, and integrative application of Myco-Root + TiO_2_ NPs enhanced proline concentration by 21.4, 22.3, and 40.8%, respectively (Table 7).

### 2.13. Malondialdehyde (MDA)

**Figure 5:**
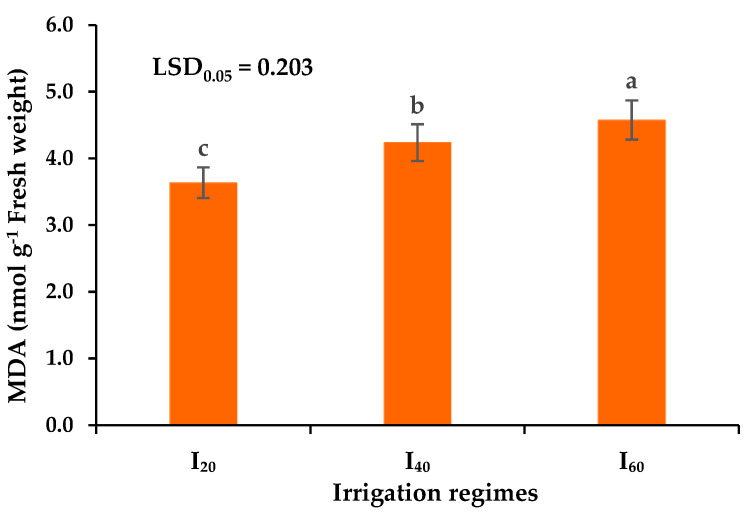
The effects of different irrigation regimes on the Malondialdehyde (MDA) content of peppermint. I_20_, I_40_, and I_60_ indicating well-watered, mild, and severe drought stress, respectively. Different letters indicate significant differences at the 5% level according to LSD’s test.

Among different irrigation regimes, the highest and lowest MDA content in peppermint leaves was observed under I_60_ and I_20_ irrigation regimes. The MDA content was enhanced by 16.5 and 25.8% in mild and severe drought stress conditions, respectively (Figure 5).

### 2.14. Antioxidant Enzymes Activity

The highest SOD, APX, and GPX activity was observed in mild drought stress (I_40_) following application of Myco-Root + TiO_2_ NPs. In comparison with non-stress conditions, the activity of APX, GPX, and SOD was enhanced by 52, 115, 124% under mild DS and 30, 66, and 42 under severe DS, respectively. Interestingly, integrative application of Myco-Root + TiO_2_ NPs enhanced the three enzyme activities by 32, 43, and 31% when compared with control (Table 7).

### 2.15. Phenolic and Flavonoid Content

The maximum phenolic (34.79 mg g^−1^) and flavonoid (17.24 mg g^−1^) content was obtained in mild drought stress (I_40_) treated with Myco-Root + TiO_2_ NPs. The lowest phenolic (25.26 mg g^−1^) and flavonoid (9.77 mg g^−1^) content was also observed in non-stress conditions (I_20_) without fertilization. In comparison with non-stress conditions, the phenolic and flavonoid content of peppermint was enhanced by 20.3, 34.4% in mild stress and enhanced by 9.4, 22.5% in severe stress, respectively. Additionally, application of Myco-Root, TiO_2_ NPs, and integrative application of Myco-Root + TiO_2_ NPs increased the phenolic content by 10.5, 9.4, and 19.2% and increased the flavonoid content by 23, 16.1, and 28.2% when compared with control, respectively (Table 7).

### 2.16. Net Income

**Figure 6:**
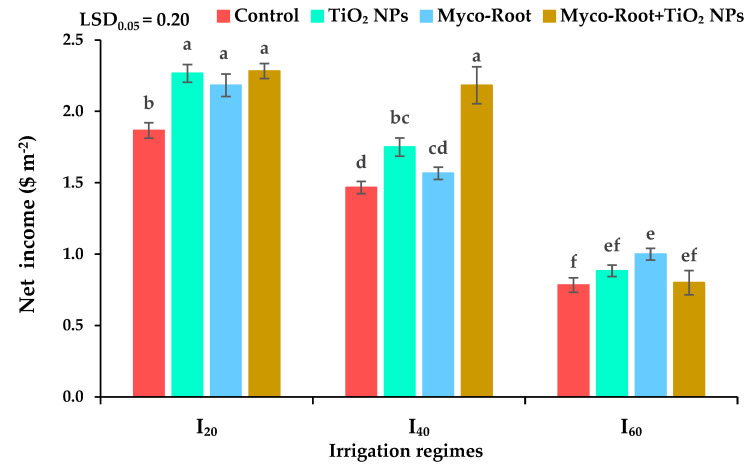
The net income of peppermint under different irrigation regimes and fertilizer sources. I_20_, I_40_, and I_60_ indicating well-watered, mild, and severe drought stress, respectively. Different letters indicate significant differences at the 5% level according to LSD’s test.

Mean comparisons showed that the net income of peppermint among different irrigation regimes and fertilizer sources was higher in well-watered conditions (I_20_) with application of Myco-Root, TiO_2_ NPs, and integrative application of Myco-Root + TiO_2_ NPs and also mild drought stress (I_40_) fertilized with integrative application of Myco-Root + TiO_2_ NPs. Under mild and severe DS conditions, the net income decreased by 19.1 and 59.5% in comparison with well-watered conditions. Additionally, application of Myco-Root, TiO_2_ NPs, and integrative application of Myco-Root + TiO_2_ NPs enhanced net income by 15.3, 19, and 28.5%, respectively, when compared with control (Figure 6).

### 2.17. Correlation

**Figure 7:**
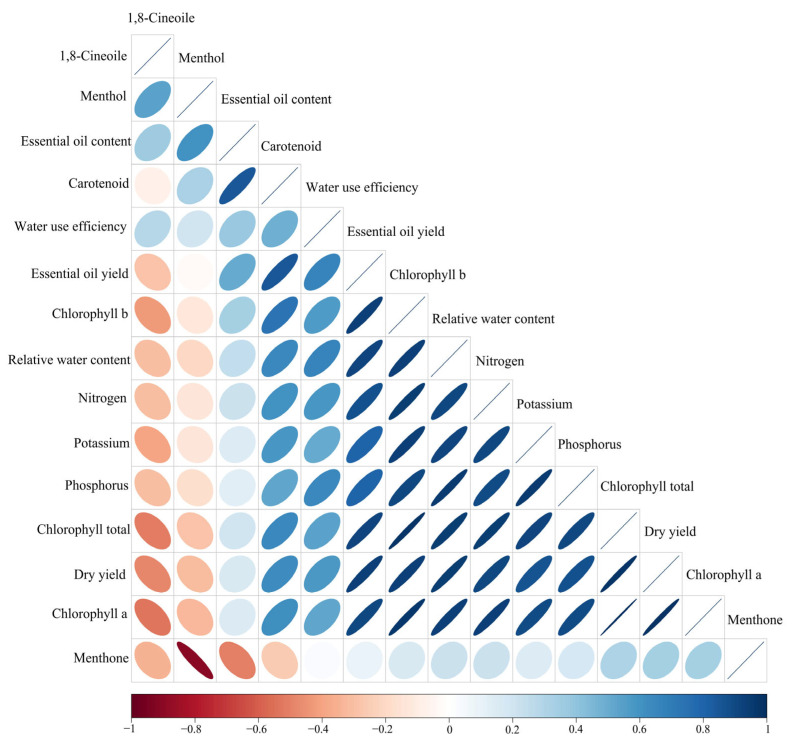
Pearson’s correlation matrix for studied traits of peppermint. Color ellipses illustrate statistically significant levels. Positive and negative correlations are shown with blue and red, respectively. The color legend on the bottom hand side of the corrplot represented intensities Pearson correlation coefficients.

Pearson’s correlation results indicated that dry yield of peppermint had a significant positive correlation with EO yield, relative water content, nitrogen, phosphorus, potassium, ch_a_, ch_b_, ch_total_, and carotenoid (r = 0.93, 0.95, 0.91, 0.87, 0.86, 0.98, 0.93, 0.98, and 0.62, respectively). In addition, water use efficiency was significantly correlated with EO yield, relative water content, nitrogen, and phosphorus concentration (r = 0.66, 0.66, 0.59, and 0.65, respectively, *p* value < 0.05). In addition, chlorophyll a had a positive and significant correlation with chlorophyll b, chlorophyll total, carotenoids, dry yield, EO yield, relative water content, nitrogen, phosphorus, and potassium concentrations (r = 0.97, 0.99, 0.61, 0.98, 0.91, 0.94, 0.94, 0.89, and 0.90, respectively). Moreover, The EO content was significantly correlated with menthol content (r = 0.60, *p* value < 0.05). Among EO constituents, negative significant correlation was observed between menthol and menthone content (r = −0.91, *p* value < 0.01) (Figure 7).

## 3. Discussion

The results of the study demonstrated the AMF inoculation rate was reduced in DS conditions. The decreasing soil moisture negatively affects spore germination and spore density, which decrease the microbial population and efficiency in soils [24]. Similarly, Ghanbarzadeh et al. [25] noted that the AMF root colonization decreased under severe DS due to reducing the availability of optimum soil moisture and insufficient supply photosynthetic carbon by the host plant for growth and spore germination of mycorrhiza fungi.

The nutrient content of peppermint decreased under DS conditions may be due to the reduction in nutrient diffusion and mass flow of nutrients under water limitation conditions [33]. In addition, the nutrient availability in the rhizosphere area depends on the activity of the soil’s microbial population. In water deficit conditions, the decreasing soil microbial activity reduced the solubility and availability of nutrients [34]. Similarly, Salehi et al. [35] showed that the concentration of nitrogen, phosphorus, and potassium of chamomile (*Matricaria chamomilla* L.) decreased under DS condition. On the other hand, the integrative application of Myco-root + TiO_2_ NPs increased the nutrient content of peppermint. The AMF–host plant symbiosis improves nutrient content in plants through enhancing surface absorbing capability as a result of the extensive hyphal network and acidification of the rhizosphere area by the release of H^+^, which increases nutrient solubility, especially bioavailable P-minerals and other micronutrients [19]. Similarly, Begum et al. [36] observed that the concentration of nitrogen, phosphorus, and potassium of tobacco (*Nicotiana tabacum* L.) increased by 48.8, 89.1, and 111.2%, respectively, with AMF inoculation. Additionally, it has been reported that the usage of TiO_2_ NPs enhanced the content of N in plants by increasing enzyme activities involved in N metabolisms and also improving transformation of inorganic N to organic N [37,38].

Our results showed that the agronomic traits (plant height and canopy diameter) and dry yield of peppermint reduced in mild and severe DS conditions. The decrease in plants productivity under DS conditions was attributed to the negative effects of water scarcity on the cell division and elongation, reducing stomata conductivity and CO_2_ uptake as a result of closing the stomata, and the increase of lipid peroxidation of membranes which decrease the photosynthesis rate and productivity of plants [39]. Similarly, Govahi et al. [40] indicated that the sage productivity decreased under DS conditions due to chloroplast destruction and decreaseing photosynthesis efficiency. On the other hand, the integrative application of Myco-Root + TiO_2_ NPs enhanced the peppermint’s agronomic traits such as plant height and canopy diameter as well as plant productivity under well-watered and DS conditions. Since there was a significant positive correlation between dry yield of peppermint and nutrient content (N, P, and K) and chlorophylls content (Figure 7), the higher productivity of peppermint under co-application of Myco-Root + TiO_2_ NPs was attributed to the increasing nutrient accessibility, which leads to increasing leaf area, chlorophyll formation, and photosynthesis capacity [30,41]. Moreover, it seems that the increasing plant height through integrative application of Myco-Root + TiO_2_ enhanced the photosynthesis rate by improving the absorption of sunlight, which led to increasing the agronomic traits and the plant’s productivity. Results of previous studies showed that shoot dry matter of coriander (*Coriandrum sativum* L.) increased by 92.3% with AMF inoculation [42]. Another study on thyme (*Thymus vulgaris* L.) showed that the shoot dry matter yield was enhanced by 56.1%, by foliar application of TiO_2_ NPs [43].

The EO content and main EO constituents of peppermint such as menthol, 1,8-cineole, and *neo*-menthol were enhanced in mild drought stress. In DS conditions, the closing stomata reduced absorption of CO_2_ and the photosynthesis rate of plants. By closing stomata, the NADPH+H^+^ concentration increases significantly in plant cells in comparison with NADP^+^, which can act as an inhibitor of photosynthesis. In this way, the biosynthesis of EO compositions conditions through usage the NADPH+H^+^ increases plant efficiency under DS conditions [11]. Similarly, Abbaszadeh et al. [12] noted that EO yield and composition of *Rosmarinus officinalis* L., such as α-pinene and camphene of rosemary, increased by 31.6, 17.8, and 38.2% in mild water stress. Furthermore, integrative application of Myco-Root + TiO_2_ NPs improved EO quali-quantitative of peppermint under DS conditions. The integrative application of the mentioned fertilizer can improve the EO content and its quality in two different ways: (i) Direct effects: in this case, the EO biosynthesis was enhanced due to increasing the accessibility of nutrients which play an important role in EO precursor compositions as well as intermediate compounds such as acetyl coenzyme A, NADPH, and ATP [44]. (ii) Indirect effects: in this case, the increasing photosynthesis rate as a result of higher accessibility of nutrients and modulating the negative effects of drought stress enhanced carbohydrates’ productivity for the growth of cells and production of EO secreting glands, and in this way, it will be able to increase EO content in the plant [45]. Similarly, Karagiannidis et al. [46] noted that inoculation with AMF increased the EO content in mint (*Mentha viridis* L.) and oregano (*Origanum onites* L.) plants. It has been reported that the EO content, yield, and main constituents of sage such as *cis*-thujone, camphor, and 1,8 cineole increased by 75, 174.5, 87.5, 30.8, and 123.1%, respectively, after foliar application of TiO_2_ [47].

Additionally, the EO yield of peppermint was enhanced in mild drought stress (I_40_) treated by Myco-Root + TiO_2_ NPs. The EO yield of peppermint is calculated from the dry yield and the EO productivity and has a direct relationship with the two mentioned factors (Figure 7). Therefore, the increasing EO yield of peppermint could be explained by the role of the integrative application of Myco-Root + TiO_2_ NPs in increasing dry yield (by increasing a plant’s yield components such as canopy diameter, etc.) and EO productivity under DS conditions.

The content of Ch_a_, Ch_b_, and Ch_total_ of peppermint reduced under mild and severe DS due to the decomposition of chlorophylls as a result of the higher activity of ROS and lipid peroxidation under stressful conditions [48]. Jahani et al. [49] concluded that the total chlorophyll concentration of peppermint reduced by 9.1 and 31.4% in moderate and severe DS conditions due to increased photo oxidation of chlorophyll and prevention of chlorophyll biosynthesis as a result of chloroplast breakdown. Bhusal et al. [50] reported that the decreasing chlorophyll content under drought stress conditions could be due to stomata closing and also inhibiting the biosynthesis of chloroplast proteins. In contrast, the carotenoid concentration was enhanced in mild drought stress. Oxidative damage of drought stress in plant tissues is modulated by the activity of enzymatic and non-enzymatic antioxidant systems. The non-enzymatic antioxidants include carotenoids (xanthophylls and carotenes), alpha-tocopherol, and ascorbates, which improve plant tolerance in the face of stressful conditions by decreasing the activity of ROS compounds [51]. The integrative application of Myco-Root + TiO_2_ NPs enhanced the chlorophylls and carotenoid concentration under DS conditions. The chlorophylls, found in the chloroplast of photosynthesizing plants, consist of two parts, including porphyrin head and a long hydrocarbon with a phytol sequence [52]. Porphyrin is composed of four nitrogen-containing pyrrole rings. The complement of the chlorophyll molecule is a Mg ion that forms a chelate with four nitrogen atoms in the center of the ring. Since the chlorophyll and carotenoid concentration have a significant and positive correlation with nutrient content (Figure 7), the enhancement of chlorophylls content could be attributed to the higher nutrient accessibility by integrative application of Myco-Root + TiO_2_ NPs.

Relative water content (RWC) of plants refers to the state of water content in plant cells, especially under DS conditions [53]. The reduction in RWC under DS conditions was due to decreasing the absorption rate of water by roots and also increasing transpiration rate by leaves. Interestingly, separate application of Myco-root and integrative application with TiO_2_ NPs enhanced the RWC content of peppermint under drought stress conditions. The symbiotic association between AMF and host plant roots improves the hydraulic conductivity of water into the roots, which will ultimately increase the relative water content in plant cells. Penetration of fungal hyphae into the root cortex and endoderm provides a path of low resistance across the root for the movement of water, and as a result, water will encounter less resistance across the root until it reaches the xylem [23]. Similarly, Gholinezhad et al. [54] determined that the RWC content of sesame (*Sesamum indicum* L.) decreased by 23 and 25% in moderate and severe DS conditions, while inoculation of the plant with two AMF species (*Funneliformis mosseae* and *Rhizophagus irregularis*) increased the RWC content by 35 and 26%, respectively.

The content of proline and MDA was enhanced in mild and severe DS conditions due to the role of ROS compounds in increasing lipid peroxidation of the cell membrane and releasing various aldehydes, such as MDA [55]. In these conditions, the increasing osmotolerant metabolites, such as proline, decrease osmotic potential and maintain turgescence pressure of plant cells, which reduces the negative effects of ROS compounds and lipid peroxidation and enhances stabilization of the membranes [56]. Similarly, Javanmard et al. [57] noted that the proline content of *Lallemantia iberica* was enhanced under moderate and severe DS conditions. Additionally, integrative application of Myco-Root + TiO_2_ NPs enhanced the proline content, especially under drought stress conditions. Since proline is one of the amino acid compounds and nitrogen is one of the essential elements in the structure of amino acids, the higher proline concentration by integrative application of Myco-Root + TiO_2_ NPs could be attributed to the availability of N through symbiotic association between AMF–host plants and also the role of TiO_2_ NPs in the conversion of inorganic N to organic N [58,59].

The antioxidant enzymes’ activities increased sharply under DS conditions. The higher activity of SOD, APX, and GPX enzymes under drought stress conditions play an important role in scavenging ROS compounds and decreasing lipid peroxidation, which leads to stabilization of the cell structure and an improvement in plant growth [60]. Additionally, the integrative application of Myco-Root + TiO_2_ NPs enhanced antioxidant enzymes’ activity. Since enzymes are protein compounds, the higher activity of enzymes by application of Myco-Root + TiO_2_ NPs was due to the accessibility of nutrients, especially nitrogen [57]. Similarly, foliar spraying of TiO_2_ NPs increased the antioxidant enzymes’ activities, such as CAT and APX in Moldavian balm and peppermint [29,61]. Furthermore, Begum et al. [62] showed that inoculation with AMF increased the antioxidant enzymes’ activities (SOD, CAT, POD, and APX) in Maize under drought stress conditions.

Phenolic and flavonoid compounds play a crucial role in the formation of various biomolecules, protecting plants against stresses. Plants produce the ROS when exposed to the stress condition, and it seems that the biosynthesis of antioxidants, flavonoids, and secondary metabolites plays an important role in protecting plant cells for detoxifying ROS and also improving protein and amino acid stabilization [63]. It is worth noting that integrative application of Myco-Root + TiO_2_ NPs, especially under mild and severe DS, improved phenolic and flavonoid content of the peppermint plant, which was attributed to enhancing nutrient availability that enhances the activity of enzymes involved in the biosynthesis of phenolic and flavonoid compounds [64]. Similarly, Shoarian et al. [65] noted that the application of TiO_2_ NPs enhanced the phenolic and flavonoid compounds of *Lallemantia iberica* under drought stress conditions. In addition, Rashidi et al. [66] reported that the content of phenolic compounds in flowers of *Ipomoea purpurea* L. enhanced by 50%, 55.8%, and 71%, respectively, after colonization with *Funneliformis mosseae*, *Rhizoglomus fasciculatum,* and *Rhizoglomus intraradices*.

Integrative application of Myco-Root + TiO_2_ NPs under mild DS enhanced the WUE of peppermint. These results indicated that the integrative application of the mentioned fertilizers enhanced the crop productivity per unit of water used as a result of improving nutrient availability, chlorophyll formation, and photosynthesis rate as well as decreasing the negative impacts of DS on the plant cells. In addition, the integrative application of Myco-Root + TiO_2_ NPs enhanced net income and economic value of peppermint plants through increasing the dry yield, EO content, and EO yield under DS conditions. Amiri et al. [67] noted that inoculation with AMF under moderate water stress on rose geranium (*Pelargonium graveolens* L.) increased water use efficiency. Our previous study results showed that integrative application of AMF + TiO_2_ increased water use efficiency of sage by 34.7% [13].

## 4. Materials and Methods

### 4.1. Study Site

A field study was conducted from 2019 to 2020 at the research farm of Maragheh University, Iran. The soil physico-chemical properties (depth of 0–30 cm) and climatic data of the experimental area are presented in Table 8 and Table 9, respectively.

### 4.2. Experiment Design and Crop Management

The experiment was conducted with a two-factor split-plot design with three replications. The main factor was three irrigation regimes which included 20% (I_20_), 40% (I_40_), and 60% (I_60_) maximum allowable depletion (MAD) percentage of the soil’s available water (SAW) as normal irrigation, mild DS, and severe DS, respectively. Moreover, the sub-factor was the application of fertilizer sources including no-fertilizer as a control, TiO_2_ NPs (100 mg L^−1^), arbuscular mycorrhizal fungi (Myco-Root) inoculation, and integrative application of Myco-Root and TiO_2_ (Myco-Root + TiO_2_ NPs). Each plot contains 5 rows of peppermint with 3 m length. Further, the distance of between rows was set to be 40 cm. Peppermint seedlings were sown by hand on the 6th and 10th day of April in 2019 and 2020, respectively. The first irrigation was performed immediately after sowing. In Myco-Root treatment, 70 g of soil including 1000 spores (*Funneliformis mosseae*) per 10 g soil obtained from Zist Fanavar Pishtaz Varian Company, Karaj, Iran (with the commercial name Myco-Root) was added to each hole at planting.

### 4.3. Implementation of Irrigation Regimes

The drought stress regimes were applied one month after sowing. The soil moisture was continuously measured with a TDR (Model German, FM-Trime) probe at depth of 0 to 30 cm. To determination of the irrigation depth used the following equations [11]:

SAW = (θ_fc_ − θ_pwp_) × d × 100 (1)

I_d_ = SAW × P (2)

I_g_ = [I_d_ × 100]/Ea (3)

in which θ_fc_ and θ_pwp_ are the soil field capacity (27.1%) and soil permanent wilting point (13.7%), respectively. D is the soil layer depth (cm), I_d_ is the depth of net irrigation, P is the fraction of SAW (20%, 40%, and 60%) which must be depleted from the root zone, Ig is a gross irrigation depth, and Ea is the efficiency of irrigation that was considered as 65% [40]. The average of the final volume water used in irrigation treatments of I_20_, I_40_, and I_60_ in both years was calculated as 1160, 870, and 580 L (=1.16, 0.87, and 0.58 m^−3^), respectively.

### 4.4. Synthesis of TiO_2_ Nanoparticle

Titanium dioxide was synthesized based on the previous method reported by Gohari et al. [29]. Firstly, an amount of desirable titanium isopropoxide was stirred and hydrolyzed under cold conditions (0 °C) to form the TiO(OH)_2_ white precipitate. The TiO(OH)_2_ precipitate was washed with distilled water. Subsequently, TiO(OH)_2_ was dissolved in nitric acid to produce a clear and homogeneous titanyl nitrate (TiO(NO_3_)_2_) solution. The TiO(NO_3_)_2_ was saved in a 250 mL beaker with a urea solution (molar ratio of 1:1). The obtained solution was placed into the muffle furnace for 2 h at 400 °C to produce solid titanium dioxide, and then it was kept inside the vacuum oven until usage. Before foliar spraying, TiO_2_ nanoparticles were dissolved in distilled water and then mixed with a mechanical stirrer for 2 h at 35 °C.

**Figure 8:**
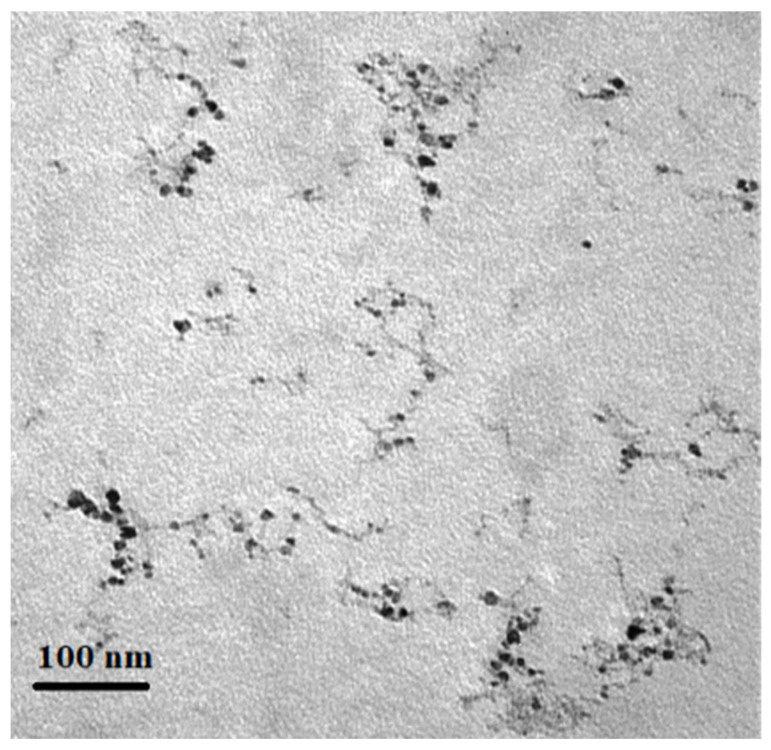
TEM image of TiO_2_ nanoparticles.

**Figure 9:**
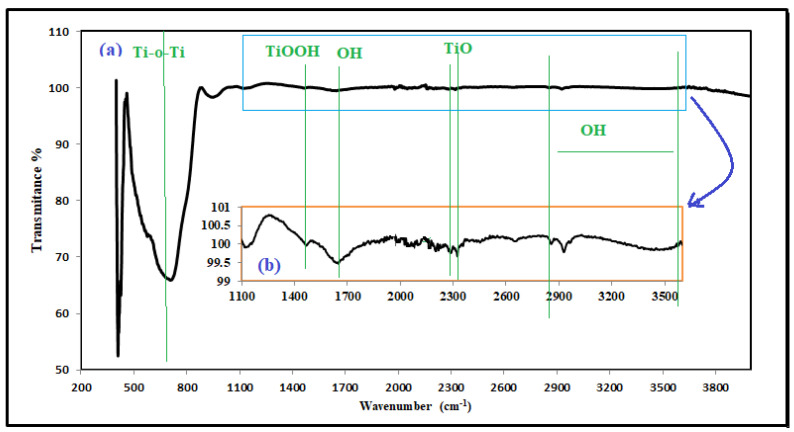
(**a**) TiO_2_ FT−IR spectrum and (**b**) FT−IR scale up from 1100 to 3500 cm^−1^.

Transmission electron microscopy (TEM) (Figure 8) and FT-IR spectrum analysis (Figure 9) was performed by a Zeiss EM-90 instrument at 80 kV tension and Perkin Elme-Spectrum Two FT-IR Spectrometer instrument, respectively. In addition, the chemical composition of the produced TiO_2_ NPs was determined using the FT-IR spectrum (Figure 9a,b). A broad peak was seen between 460 and 870 cm^−1^, which was consistent with Ti-O-Ti stretching vibration bonds [68] (Figure 9a). The broad bands at 2700–3600 and 1625 cm^−1^ were identified as the result of stretching and bending vibrations of O-H groups of adsorbed water on the surface of TiO_2_, which are crucial for photocatalytic activity, respectively. The Ti-O modes were associated with the peak at 1473 cm^−1^. The stretching vibration of TiOO-H is represented by the double absorption peak at 2288 and 2324 cm^−1^ [69,70] (Figure 9b).

**Figure 10:**
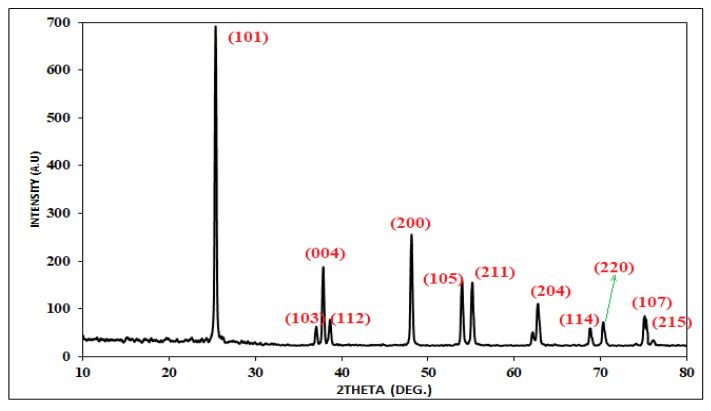
XRD patterns of the TiO_2_ nanoparticles.

**Figure 11:**
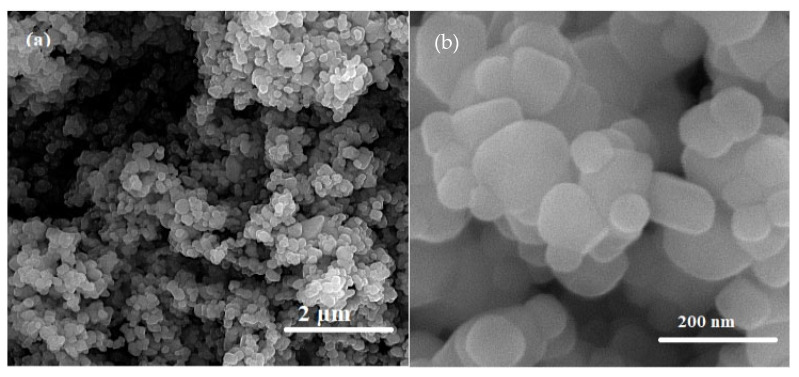
SEM images of TiO_2_ NPs at different magnifications (**a**,**b**).

In addition, the XRD and SEM analyses were conducted at the Central Laboratory of the University of Tabriz, Iran using Tongda-TD-3700 and VWGA3 TESCAN instruments, respectively (Figure 10 and Figure 11). X-ray diffraction (XRD) was used to confirm that TiO_2_ nanoparticles were crystalline and had a certain shape (Figure 10). The characteristic peaks of TiO_2_ in the anatase phase were connected to all of the diffraction peaks in the XRD spectrum (JCPDS file 73–1764). In fact, there were diffraction peaks at 2θ = 25.4°, 37.16°, 37.96°, 38.7°, 48.18°, 53.94°, 55.24°, 62.86°, 68.94°, 70.44°, 75.4°, and 76.3°, and they were accurately indexed to the appropriate tetragonal crystal faces (101), (103), (004), (112), (200), (105), (211), (204), (114), (220), (107), and (211), respectively [69,71,72]. The size (D_hkl_) of the crystal domains was determined by using the widths at half maximum height (B) in the XRD peak’s broadening. This was achieved with the Debye-Scherrer equation:

(4) $$D_{\mathrm{hkl}}=\frac{k\lambda }{\mathrm{Bcos}\theta }$$

where λ is the X-ray beam’s wavelength (1.5406 Å), θ is the Bragg angle, and K is a constant attributed to the shape of the domain, that is about 0.9. The average size of crystallite in the TiO_2_ nanoparticles that were made was estimated to be about 18 nm. The SEM was used to study the morphology of the synthesized NPs, which are depicted in Figure 11a,b for TiO_2_ at two different magnifications. In the SEM image of TiO_2_, some agglomeration was also seen, which may be caused by calcination NPs. Synthesized TiO_2_ NPs were discovered to be spherical in shape.

### 4.5. Measurements

#### 4.5.1. Agronomic Traits, Dry Yield, and Water User Efficiency

Before harvesting, the agronomic traits of peppermint were measured in ten plants of each plot randomly. In both years, the aerial parts of peppermint were harvested randomly at the full flowering stage from a 1.4 m^−2^ area. In order to determine the dry yield of peppermint, the harvesting samples were kept at room temperature without direct sunlight for two weeks. Additionally, the water use efficiency (WUE) was calculated as [73]:

WUE (g m^−3^) = Dry yield/Final volume water used in irrigation treatments (5)

#### 4.5.2. Essential Oil Analysis

##### Essential Oil Extraction

The peppermint EO was extracted from 40 gr of dried aerial parts of peppermint mixed with 400 mL that hydro-distillated for 3 h by a Clevenger-type apparatus. After extracting EO, the samples were dried by adding sodium sulfate and kept at 4 °C for chemical analysis. The EO yield of peppermint was calculated based on the following equation [74]:

Essential oil (%) = [EO extracted by hydro distillation/40 gr dried aerial parts] × 100(6)

EO yield (g m^−2^) = EO content × dry yield of aerial part (7)

##### GC/MS and GC-FID Analysis

The peppermint EO compounds were identified using a gas chromatograph instrument (Agilent 7990 B, USA) equipped to a 5988A mass spectrometer with HP-5MS capillary column (length 30 m, 5% phenylmethyl polysiloxane, 0.25 mm inner diameter, and 0.25 μm film thickness). Briefly, the first temperature of the oven reached 60 °C in 5 min, and was subsequently at a rate of 3 °C/min reached in 240 °C and maintained for 20 min. The carrier gas was helium, which was set up with a flow rate of 1 mL/min. The injector and detector temperatures were set up at 230 and 240 °C, respectively. The injector was adjusted based on the split ratio of 1:30. Mass scan range and ionization temperatures were 40–400 *m*/*z* and 220 °C, respectively. The mass spectra were recorded using an electron impact ionization technique (ionization energy of 70 eV). In order to compute the linear retention indices (RIs), a mixture of n-alkane series was co-injected with the essential oil into the GC instrument based on the above temperature conditions [5]. For identification of volatile ingredients, their retention times compared with those retention indices that were reported in reference libraries [75] or through comparison of their mass spectra with data in the NIST 05 database. Volatile components were separated using an Agilent 7990B gas chromatography instrument (USA) with a capillary column VF-5MS (0.50 μm f.t., 30 mL, 0.25 mm i.d., 5% phenyl methylpolysiloxane) and flame ionization detector (FID). The oven temperature program was the same as the above analytical conditions. The injection volume of essential oil was 1 µL that was diluted in an *n*-hexane solution (1:100). The peak area normalization method was also used for calculating the percentage compositions of essential oil [74].

#### 4.5.3. Nutrient Content

The nitrogen (N) content of peppermint leaves was measured based on the Kjeldahl method [76]. In order to determine the phosphorus (P) and potassium (K) concentration of peppermint, 1 gr of dried leaves of peppermint was put into the porcelain crucible and placed in a muffle furnace. The temperature of the muffle furnace reached 550 °C gradually and was maintained for 8 h until a white ash formed. After cooling the muffle furnace, the samples were dissolved in 5 mL hydrochloric acid (HCL) 2N. After 15 min, the samples filtered through filter paper (Whatman No. 42), were brought to the volume (50 mL), and stood for 30 min. The concentration of K and P was determined by flame photometry (Jenway PFP7C) and the yellow color method (colorimetric method by Ammonium Vanadate/Molybdate) using a spectrophotometer at 470 nm absorption spectra, respectively [77].

#### 4.5.4. Relative Water Content (RWC)

For the determination of RWC in peppermint leaves, firstly, the fresh weight of 10 fully developed leaves was measured (LFW). Then, the samples were kept in distilled water at 4 °C and measured leaf turgor weight after 24 h (LTW). After that, the leaves dried in the oven at 70 °C for 48 h and the leaf dry weight (LDW) was weighed [78]. Finally, RWC content was calculated by the following formula:

(8) $$\mathrm{RWC}\;(\%)=\frac{\mathrm{LFW}-\mathrm{LDW}}{\mathrm{LTW}-\mathrm{LDW}}\times 100$$

#### 4.5.5. Chlorophyll and Carotenoid

In order to quantify the content of chlorophyll a (Ch_a_), b (Ch_b_) and carotenoid, 0.5 gr of peppermint fresh leaves was digested in liquid nitrogen and mixed with 10 mL of 80% acetone. Samples centrifuged at 10,000 rpm and supernatant after 10 min were transferred to a new tube, and the absorbance was read at 645, 663, and 470 nm spectrophotometrically (UV-1800, Shimadzu, Japan). After that, the content of photosynthetic pigments was calculated by following equations [79]:

Ch_a_ = [(12.7 × Abs_663_) − (2.69 × Abs_645_)] (9)

Ch_b_ = [(21.5 × Abs_645_) − (5.1 × Abs_663_)] (10)

Carotenoid = [(1000 × Abs_470_) − (1.82 × Ch_a_ − 85.02Ch_b_)/198] (11)

#### 4.5.6. Malondialdehyde (MDA)

For determination of Malondialdehyde (MDA) concentration, firstly, 0.1 g of peppermint fresh leaves sample was extracted using 1 mL 0.1% TCA (trichloroacetic acid) and centrifuged at 12,000 rpm. After 15 min, the supernatant was mixed with 4 mL of reaction mixture including 20% TCA and 0.67% 2-thiobarbituric acid (TBA). After that, the sample was heated in a water bath at 95 °C for 15 min and then quickly cooled down in an ice bath for 10 min and centrifuged at 10,000 rpm for 5 min at 4 °C. Finally, the absorbance was recorded at 532 and 600 nm spectrophotometrically (UV-1800, Shimadzu, Japan), and the MDA concentration was represented as nmol g^−1^ FW [80].

#### 4.5.7. Proline

Firstly, we took a 0.5 g sample of fresh peppermint leaves after being digested in liquid nitrogen, mixed with 10 mL 3% sulfosalicylic acid and then centrifuged at 12,000 rpm. After 10 min, 2 mL of the separated supernatant and the samples were put into a tube and mixed with 2 mL of acid-ninhydrin and 2 mL of glacial acetic acid. The obtained mixtures were heated in a water bath for 1h at 100 °C and then placed on an ice bath. Then, 4 mL of toluene was added to the samples and vortexed for 20 s. Finally, absorbance was read at 520 nm spectrophotometrically, and the proline content was represented as µmol g^−1^ of fresh weight [81].

#### 4.5.8. Antioxidant Enzyme Activity

##### Guaiacol Peroxidases (GPX) Activity

In order to prepare the reaction mixture, firstly, we mixed a hydrogen peroxide (70 mM), phosphate buffer (50 mM) and 10 mM of guaiacol and then added 20 μL of enzyme extract to the reaction mixture. The absorbance was read at 420 nm for 180 s and represented as μmol min^−1^ mg^−1^ protein [82].

##### Ascorbate Peroxidase (APX) Activity

The reaction mixture for APX was prepared with a mixture of 0.1 mM H_2_O_2_, potassium phosphate buffer (50 mM, pH = 7), 0.5 mM ascorbic acid, and 0.1 mM EDTA. Then, 150 μL of enzyme extract was added to the reaction mixture. After that, the absorbance was read at 290 nm (coefficient of absorbance = 2.8 mmol^−1^ cm^−1^) [83].

##### Superoxide Dismutase (SOD) Activity

The reaction mixture for the determination of SOD included L-methionine (12 mM), potassium phosphate buffer (50 mM, pH = 7.8), sodium carbonate (50 mM), 2 μM of riboflavin, 0.1 mM EDTA, and 75μM of nitro blue tetrazolium (NBT). A 100 μL amount of enzyme extract was added to the reaction mixture and kept in 25 °C under illumination. After 15 min, absorbance was read at 560 nm and represented as μmol min^−1^ mg^−1^ protein [84].

#### 4.5.9. Total Phenolic and Flavonoid Content

The total phenolic and flavonoid content of peppermint was determined based on the previous method, which was reported by Singleton and Rosi [85] and Nagy and Grancai [86].

#### 4.5.10. Determination of Root Colonization

To specify the AMF colonization rate, firstly, the collected root samples were washed with water for removing extra particles and then cut into pieces of 1 cm length. In order to eliminate cytoplasmic contents from cells, which facilitates staining, root pieces were cleared in hot 10% KOH for 10 min. After rinsing three times with distilled water, the root sample was acidified in 2% HCL for 15 min at room temperature and stained in a hot 20% lactophenol cotton blue solution for 15 min [87,88]. After coloring, the AMF colonization percentage was determined using the grid line intersection method, which was described by Giovannetti and Mosse [89].

### 4.6. Statistical Analysis

The normality and homoscedasticity of data regarding the morphological and physiological traits were verified using the Kolmogorov–Smirnov and Levene test, respectively. Combined variance analysis of data was carried out with SAS v9.4 (SAS Institute, Cary, NC, USA) and SPSS v25 software. The mean comparison between treatments was analyzed by the LSD test at probability levels of 0.05%. Irrigation levels and fertilizer sources were considered as fixed factors, and block and years were random factors. In addition, for drawing the Pearson correlation matrix (correlation plot) between main studied traits such as dry yield, EO content, EO yield, photosynthetic pigments, nitrogen, phosphorus, potassium, and major EO compounds (menthol, menthone, and 1,8-cineole), R software v3.2.4 was used.

## 5. Conclusions

The results of the study showed that the integrative application of Myco-Root + TiO_2_ has great potential for improving plant performance under drought stress conditions by enhancement of the nutrient uptake, chlorophyll formation, and relative water content as well as antioxidant activity. Importantly, improving essential oil quantity and quality of peppermint demonstrated another positive impact of the integrative application of Myco-Root + TiO_2_ under stressful conditions. Generally, the results of the study indicated that the integrative application of Myco-Root + TiO_2_ NPs could be suggested as a sustainable strategy for improving EO quantity and quality of peppermint under DS conditions.

## Figures and Tables

**Table 1:** The ANOVA (*p*-value) results of root colonization, nutrient content, agronomic traits, dry yield, essential oil content and yield, water use efficiency, and net income of peppermint affected by experimental factors.

Source of Variation	Root Colonization	N Content	P Content	K Content	Plant Height	Canopy Diameter	Dry Yield	EO Content	EO Yield	WUE	Net Income
Y	0.028 *	0.225 ^ns^	0.336 ^ns^	0.04 ^*^	0.98 ^ns^	0.101 ^ns^	0.167 ^ns^	0.615 ^ns^	0.199 ^ns^	0.016 *	0.141 ^ns^
I	<0.0001 **	0.022 *	0.012 *	<0.0001 **	<0.0001 **	<0.0001 **	<0.0001 **	<0.0001 **	<0.0001 **	0.041 *	<0.0001 **
F	<0.0001 **	0.003 **	0.031 *	<0.0001 **	<0.0001 **	<0.0001 **	0.025 *	0.0007 **	<0.0001 **	0.064 ^ns^	<0.0001 **
Y*I	0.196 ^ns^	0.387 ^ns^	0.052 ^ns^	0.873 ^ns^	0.072 ^ns^	0.239 ^ns^	0.334 ^ns^	0.921 ^ns^	0.580 ^ns^	0.135 ^ns^	0.561 ^ns^
I*F	<0.0001 **	0.007 **	<0.0001 **	0.04 *	0.526 ^ns^	0.720 ^ns^	<0.0001 **	0.032 *	<0.0001 **	0.012 *	<0.0001 **
Y*F	0.298 ^ns^	0.977 ^ns^	0.101 ^ns^	0.909 ^ns^	0.365 ^ns^	0.190 ^ns^	0.104 ^ns^	0.916 ^ns^	0.701 ^ns^	0.079 ^ns^	0.766 ^ns^
Y*I*F	0.217 ^ns^	0.998 ^ns^	0.839 ^ns^	0.577 ^ns^	0.754 ^ns^	0.079 ^ns^	0.743 ^ns^	0.547 ^ns^	0.317 ^ns^	0.313 ^ns^	0.408 ^ns^

Y: year; I: irrigation regimes; F: fertilizer sources. ns, * and ** indicated no significant difference, significant at 5% probability level, and significant at 1% probability level, respectively.

**Table 2:** The ANOVA (*p*-value) results of chlorophylls, carotenoids content, relative water content, antioxidant enzymes activities, proline, malondialdehyde, total phenolic, and flavonoid content of peppermint affected by experimental factors.

Source of Variation	Ch_a_	Ch_b_	Ch_total_	Carotenoid	RWC	APX	GPX	SOD	MDA	Proline	Total Phenolic Content	Total Flavonoid Content
Y	0.021 *	0.816 ^ns^	0.087 ^ns^	0.414 ^ns^	0.094 ^ns^	0.526 ^ns^	0.735 ^ns^	0.935 ^ns^	0.745 ^ns^	0.952 ^ns^	0.523 ^ns^	0.412 ^ns^
I	0.034 *	0.023 *	0.04 *	<0.0001 **	<0.0001 **	<0.0001 **	<0.0001 **	<0.0001 **	<0.0001 **	<0.0001 **	<0.0001 **	<0.0001 **
F	0.021 *	0.035 *	0.003 **	0.040 *	<0.0001 **	<0.0001 **	0.023 *	<0.0001 **	0.145 ^ns^	0.015 *	<0.0001 **	<0.0001 **
Y*I	0.452 ^ns^	0.56 ^ns^	0.014 *	0.089 ^ns^	0.299 ^ns^	0.155 ^ns^	0.058 ^ns^	0.635 ^ns^	0.091 ^ns^	0.326 ^ns^	0.287 ^ns^	0.217 ^ns^
I*F	0.014 *	0.015 *	0.02 *	0.713 ^ns^	<0.0001 **	<0.0001 **	<0.0001 **	<0.0001 **	0.085 ^ns^	<0.0001 **	<0.0001 **	0.001 **
Y*F	0.196 ^ns^	0.116 ^ns^	0.213 ^ns^	0.983 ^ns^	0.204 ^ns^	0.703 ^ns^	0.638 ^ns^	0.669 ^ns^	0.934 ^ns^	0.702 ^ns^	0.635 ^ns^	0.613 ^ns^
Y*I*F	0.632 ^ns^	0.808 ^ns^	0.320 ^ns^	0.377 ^ns^	0.628 ^ns^	0.999 ^ns^	0.999 ^ns^	0.999 ^ns^	0.955 ^ns^	0.998 ^ns^	0.999 ^ns^	0.999 ^ns^

Y: year; I: irrigation regimes; F: fertilizer sources. ns, * and ** indicated no significant difference, significant at 5% probability level, and significant at 1% probability level, respectively.

**Table 3:** The concentration of nitrogen (N), phosphorus (P), and potassium (K) of peppermint in different irrigation regimes and fertilizer sources.

Treatments		N (g kg^−1^)	P (g kg^−1^)	K (g kg^−1^)
I_20_	Control	20.03 ^e^	1.55 ^de^	20.78 ^cde^
	TiO_2_ NPs	26.75 ^a^	1.72 ^c^	22.92 ^bc^
	Myco-Root	25.48 ^b^	1.86 ^b^	25.02 ^b^
	Myco-Root + TiO_2_ NPs	26.92 ^a^	2.04 ^a^	27.80 ^a^
I_40_	Control	21.53 ^d^	1.40 ^f^	20.68 ^cde^
	TiO_2_ NPs	21.53 ^d^	1.55 ^de^	19.72 ^def^
	Myco-Root	21.50 ^d^	1.62 ^d^	22.87 ^bc^
	Myco-Root + TiO_2_ NPs	22.52 ^c^	1.61 ^d^	21.65 ^cd^
I_60_	Control	17.43 ^g^	1.28 ^g^	17.54 ^f^
	TiO_2_ NPs	18.57 ^f^	1.41 ^f^	17.95 ^f^
	Myco-Root	18.75 ^f^	1.50 ^e^	18.80 ^ef^
	Myco-Root + TiO_2_ NPs	19.03 ^f^	1.39 ^f^	18.88 ^ef^
LSD_0_._05_		0.97	0.08	2.45

I_20_, I_40_, and I_60_ indicating well-watered, mild, and severe drought stress, respectively. Different letters indicate significant differences at the 5% level according to LSD’s test.

**Table 4:** The dry yield, essential oil content, essential oil yield, and water use efficiency of peppermint in different irrigation regimes and fertilizer sources.

Treatments		Dry Yield (g m^−2^)	Essential Oil Content (%)	Essential Oil Yield (g m^−2^)	Water Use Efficiency (g m^−3^)
I_20_	Control	163.81 ^c^	1.06 ^ef^	1.73 ^c^	141.38 ^efg^
	TiO_2_ NPs	187.03 ^b^	1.15 ^de^	2.15 ^ab^	161.45 ^bc^
	Myco-Root	179.05 ^b^	1.15 ^de^	2.06 ^b^	154.57 ^bcde^
	Myco-Root + TiO_2_ NPs	195.72 ^a^	1.13 ^e^	2.21 ^ab^	168.97 ^ab^
I_40_	Control	117.62 ^f^	1.27 ^cd^	1.49 ^d^	135.42 ^g^
	TiO_2_ NPs	131.15 ^e^	1.39 ^ab^	1.83 ^c^	151.00 ^cdef^
	Myco-Root	121.35 ^f^	1.34 ^bc^	1.62 ^cd^	139.73 ^efg^
	Myco-Root + TiO_2_ NPs	154.40 ^d^	1.49 ^a^	2.30 ^a^	177.77 ^a^
I_60_	Control	78.76 ^h^	1.01 ^f^	0.79 ^f^	135.98 ^fg^
	TiO_2_ NPs	85.74 ^gh^	1.13 ^e^	0.97 ^ef^	148.02 ^cdefg^
	Myco-Root	91.17 ^g^	1.12 ^ef^	1.02 ^e^	157.40 ^bcd^
	Myco-Root + TiO_2_ NPs	82.58 ^h^	1.10 ^ef^	0.91 ^ef^	142.55 ^defg^
LSD _0_._05_		8.20	0.120	0.213	15.03

I_20_, I_40_, and I_60_ indicating well-watered, mild, and severe drought stress, respectively. Different letters indicate significant differences at the 5% level according to LSD’s test.

**Table 5:** The essential oil constituents of peppermint in different irrigation regimes and fertilizers (average of two years).

				Treatments											
No	Components	RI ^a^	LIT. RI ^b^	I_20_ Control	I_20_ TiO_2_ NPs	I_20_ Myco-Root	I_20_ Myco-Root + TiO_2_ NPs	I_40_ Control	I_40_ TiO_2_ NPs	I_40_ Myco-Root	I_40_ Myco-Root + TiO_2_ NPs	I_60_ Control	I_60_ TiO_2_ NPs	I_60_ Myco-Root	I_60_ Myco-Root + TiO_2_ NPs
1	*α*-Pinene	931	932	0.79	0.66	0.59	0.57	0.46	0.54	0.62	0.42	0.64	0.63	0.56	0.59
2	Sabinene	970	969	0.83	0.75	0.55	0.60	0.47	0.53	0.58	0.49	0.65	0.58	0.57	0.67
3	*β*-Pinene	975	974	1.10	1.08	1.04	1.04	0.83	0.90	0.96	0.80	1.14	1.05	1.04	1.08
4	Myrcene	988	988	0.69	0.35	0.33	0.44	0.28	0.30	0.29	0.25	0.34	0.33	0.33	0.38
5	3-Octanol	1000	998	0.51	0.32	0.38	0.29	0.64	0.41	0.54	0.72	0.69	0.65	0.71	0.66
6	*α*-Terpinene	1017	1014	0.14	0.14	0.16	0.15	0.10	0.14	0.11	0.09	0.18	0.18	0.12	0.14
7	Limonene	1026	1024	2.10	2.02	1.97	1.67	2.00	2.32	2.25	1.82	2.24	2.21	2.25	2.04
8	1,8-Cineole	1029	1026	6.55	6.99	7.16	6.89	7.30	7.27	7.04	7.84	6.84	7.80	7.78	7.76
9	*γ*-Terpinene	1058	1054	0.42	0.36	0.24	0.35	0.22	0.20	0.36	0.21	0.28	0.27	0.23	0.25
10	*cis*-Sabinene hydrate	1066	1065	3.00	2.85	2.81	2.92	2.57	2.64	2.64	2.79	2.68	2.74	2.78	2.78
11	Linalool	1103	1095	0.54	0.57	0.58	0.51	0.63	0.58	0.48	0.68	0.64	0.62	0.65	0.60
12	Menthone	1152	1148	20.10	20.13	13.88	17.87	16.20	12.72	13.31	12.77	13.83	18.81	15.43	13.86
13	Menthofuran	1161	1159	0.33	0	0	0.26	0	0	0.28	0	0	0.43	0	0
14	*δ*-Terpineol	1162	1162	2.83	2.78	2.44	2.53	2.23	2.14	2.29	2.81	2.05	3.18	3.35	3.04
15	*neo*-Menthol	1163	1161	3.29	3.79	4.39	4.38	3.88	4.14	4.06	4.52	3.14	3.93	3.60	4.18
16	Menthol	1175	1167	38.99	42.04	47.25	44.33	46.66	48.49	47.15	52.00	48.24	44.31	45.89	47.50
17	Terpinen-4-ol	1177	1177	0.83	0.96	1.01	1.06	1.03	1.15	1.62	1.02	0.95	1.04	1.01	1.34
18	*neo*-*iso*-Menthol	1184	1184	1.14	1.00	1.39	1.27	1.05	1.18	1.38	1.23	1.07	0.99	0.99	1.42
19	Pulegone	1236	1233	0.00	0.10	0.10	0.00	0.16	0.21	0.00	0.00	0.41	0.23	0.34	0.14
20	Piperitone	1252	1252	0.58	0.58	0.47	0.43	0.55	0.61	0.73	0.55	0.51	0.58	0.58	0.50
21	*neo*-Menthyl acetate	1273	1271	0.24	0.13	0.28	0.30	0.20	0.24	0.22	0.19	0.11	0.09	0.10	0.19
22	*p*-Menth-l-en-9-ol	1294	1294	1.96	2.27	3.26	3.03	2.30	3.09	2.97	2.29	1.93	1.95	1.29	2.76
23	*iso*-Menthyl acetate	1307	1304	0.39	0.11	0.17	0.49	0.11	0.16	0.22	0.11	0.00	0.11	0.11	0.14
24	*β*-Bourbonene	1382	1387	0.72	0.51	0.52	0.46	0.48	0.51	0.63	0.38	0.49	0.41	0.47	0.58
25	(*E*)-Caryophyllene	1416	1417	1.62	1.88	1.72	1.79	1.83	1.82	1.87	1.24	1.83	1.73	2.00	1.85
26	(*E*)-β-Farnesene	1457	1454	0.52	0.27	0.29	0.28	0.33	0.34	0.81	0.22	0.28	0.26	0.40	0.31
27	Germacrene D	1479	1484	2.73	2.19	1.78	2.04	1.94	1.91	2.51	1.47	1.92	1.73	2.19	2.02
28	Elixene	1494	1492	1.02	0.35	0.31	0.32	0.32	0.37	0.37	0.31	0.36	0.30	0.46	0.35
29	Viridiflorol	1589	1592	1.08	1.24	1.29	1.82	1.33	1.44	1.81	1.32	1.40	1.01	1.42	1.33
	Total identified (%)			95.05	96.40	96.36	98.05	96.07	96.32	98.09	98.51	94.82	98.16	96.63	98.46
	Grouped compounds (%)														
	Monoterpene hydrocarbons			6.08	5.35	4.89	4.81	4.36	4.92	5.17	4.07	5.46	5.26	5.10	5.15
	Oxygenated monoterpenes			80.76	84.30	85.18	86.24	84.85	84.61	84.38	88.78	82.39	86.82	83.89	86.21
	Sesquiterpene hydrocarbons			6.62	5.19	4.62	4.88	4.90	4.95	6.18	3.62	4.88	4.42	5.51	5.11
	Oxygenated sesquiterpenes			1.08	1.24	1.29	1.82	1.33	1.44	1.81	1.32	1.40	1.01	1.42	1.33
	Others			0.51	0.32	0.38	0.29	0.64	0.41	0.54	0.72	0.69	0.65	0.71	0.66

^a^ RI, linear retention indices on DB-5 MS column, experimentally determined using homologue series of n-alkanes. ^b^ Relative retention indices taken from Adams. I_20_, I_40_, and I_60_ indicating well-watered, mild, and severe drought stress, respectively.

**Table 6:** Chlorophyll a, b, total, and relative water content of peppermint in different irrigation regimes and fertilizer sources.

Treatments		Chlorophyll a (mg g^−1^ Fresh Weight)	Chlorophyll b (mg g^−1^ Fresh Weight)	Chlorophyll Total (mg g^−1^ Fresh Weight)	Relative Water Content (%)
I_20_	Control	3.35 ^cd^	0.87 ^c^	4.22 ^c^	78.85 ^e^
	TiO_2_ NPs	4.68 ^ab^	1.31 ^ab^	5.99 ^ab^	83.90 ^c^
	Myco-Root	3.95 ^bc^	1.19 ^bc^	5.14 ^b^	87.05 ^b^
	Myco-Root + TiO_2_ NPs	5.13 ^a^	1.67 ^a^	6.80 ^a^	90.12 ^a^
I_40_	Control	2.17 ^e^	0.83 ^cd^	3.00 ^d^	69.90 ^g^
	TiO_2_ NPs	2.67 ^de^	1.00 ^bc^	3.67 ^cd^	75.82 ^f^
	Myco-Root	2.68 ^de^	1.02 ^bc^	3.70 ^cd^	77.30 ^ef^
	Myco-Root + TiO_2_ NPs	2.91 ^de^	1.08 ^bc^	3.98 ^c^	80.95 ^d^
I_60_	Control	0.90 ^f^	0.34 ^e^	1.24 ^e^	61.13 ^i^
	TiO_2_ NPs	0.94 ^f^	0.39 ^e^	1.33 ^e^	68.08 ^h^
	Myco-Root	1.08 ^f^	0.44 ^de^	1.52 ^e^	71.40 ^g^
	Myco-Root + TiO_2_ NPs	0.91 ^f^	0.44 ^de^	1.35 ^e^	67.63 ^h^
LSD _0_._05_		0.97	0.40	0.87	1.80

I_20_, I_40_, and I_60_ indicating well-watered, mild, and severe drought stress, respectively. Different letters indicate significant differences at the 5% level according to LSD’s test.

**Table 7:** Antioxidant enzymes activity, proline, and total phenolic and flavonoid content of peppermint leaves in different irrigation regimes and fertilizer sources.

Treatments		APX (μmol min^−1^ mg^−1^ Protein)	GPX (μmol min^−1^ mg^−1^ Protein)	SOD (μmol min^−1^ mg^−1^ Protein)	Proline (µmol g^−1^ Fresh Weight)	Total Phenolic Content (mg g^−1^)	Total Flavonoid Content (mg g^−1^)
I_20_	Control	0.203 ^j^	0.337 ^l^	2.73 ^l^	0.52 ^k^	25.26 ^k^	9.77 ^k^
	TiO_2_ NPs	0.227 ^i^	0.422 ^j^	3.03 ^j^	0.61 ^j^	26.73 ^i^	11.19 ^j^
	Myco-Root	0.243 ^h^	0.388 ^k^	2.93 ^k^	0.63 ^j^	27.77 ^h^	13.17 ^h^
	Myco-Root + TiO_2_ NPs	0.245 ^h^	0.473 ^i^	3.48 ^i^	0.66 ^i^	30.27 ^f^	13.44 ^g^
I_40_	Control	0.312 ^f^	0.773 ^e^	6.17 ^d^	1.01 ^h^	31.03 ^e^	14.21 ^f^
	TiO_2_ NPs	0.350 ^b^	0.882 ^b^	6.62 ^b^	1.39 ^g^	34.13 ^b^	15.85 ^c^
	Myco-Root	0.330 ^d^	0.832 ^c^	6.35 ^c^	1.46 ^f^	32.46 ^d^	16.60 ^b^
	Myco-Root + TiO_2_ NPs	0.402 ^a^	1.035 ^a^	8.13 ^a^	1.61 ^d^	34.79 ^a^	17.24 ^a^
I_60_	Control	0.245 ^h^	0.508 ^h^	3.69 ^h^	1.55 ^e^	26.34 ^j^	12.35 ^i^
	TiO_2_ NPs	0.315 ^e^	0.695 ^g^	4.58 ^f^	1.76 ^b^	29.57 ^g^	15.14 ^d^
	Myco-Root	0.308 ^g^	0.732 ^f^	4.18 ^g^	1.66 ^c^	31.02 ^e^	14.89 ^e^
	Myco-Root + TiO_2_ NPs	0.343 ^c^	0.803 ^d^	4.85 ^e^	2.07 ^a^	33.41 ^c^	15.90 ^c^
LSD _0_._05_		0.0032	0.0118	0.08	0.029	0.25	0.119

I_20_, I_40_, and I_60_ indicating well-watered, mild, and severe drought stress, respectively. Different letters indicate significant differences at the 5% level according to LSD’s test.

**Table 8:** Physico-chemical properties of field soil (average of two years).

Soil Texture	Sand (%)	Silt (%)	Clay (%)	OM (g kg^−1^)	EC (ds.m^−1^)	pH	FC (%)	PWP (%)	CEC (Cmolc kg^−1^)	N (g kg^−1^)	P (mg kg^−1^)	K (mg kg^−1^)
Sandy clay loam	56.3	16.3	27.4	8.1	1.17	7.73	27.1	13.7	26.5	0.87	9.7	563.85

OM: organic matter, FC: field capacity, PWP: permanent wilting point, CEC: cation exchange capacity.

**Table 9:** Monthly average temperature and total monthly precipitation in 2019 and 2020 growing seasons and long-term averages in the experimental area.

Year	April	May	June	July	August	September
Monthly average temperature (°C)						
2019	10.4	18.5	25.7	27.6	27.8	22.1
2020	11.8	19.1	24.2	28.0	25.1	23.8
2-year mean	11.1	18.8	25.0	27.8	26.5	23.0
10-year mean	12.6	18.2	24.1	28.1	27.5	22.7
Total monthly precipitation (mm)						
2019	51.3	37.8	4.2	0.0	0.0	0.0
2020	63.3	12.0	2.6	0.1	1.2	0.0
2-year mean	57.3	24.9	3.4	0.1	0.6	0.0
10-year mean	44.8	20.6	1.7	0.5	0.4	2.0

## Data Availability

The datasets generated and analyzed during the current study are available from the corresponding author upon reasonable request.
